# Changes in functional connectivity in newly diagnosed self‐limited epilepsy with centrotemporal spikes and cognitive impairment: An MEG study

**DOI:** 10.1002/brb3.2830

**Published:** 2022-11-21

**Authors:** Yihan Li, Jinan Chen, Jintao Sun, Ping Jiang, Jing Xiang, Qiqi Chen, Zheng Hu, Xiaoshan Wang

**Affiliations:** ^1^ Department of Neurology The Affiliated Brain Hospital of Nanjing Medical University Nanjing Jiangsu China; ^2^ MEG Center, Division of Neurology Cincinnati Children's Hospital Medical Center Cincinnati Ohio USA; ^3^ MEG Center Nanjing Brain Hospital Nanjing Jiangsu China; ^4^ Department of Neurology Nanjing Children's Hospital Nanjing Jiangsu China

**Keywords:** cognitive function, functional connectivity, magnetoencephalography, multifrequency bands, self‐limited epilepsy with centrotemporal spikes

## Abstract

**Purpose:**

Our purpose was to explore the relationship between cognitive impairment and neural network changes in patients newly diagnosed with self‐limited epilepsy with centrotemporal spikes (SeLECTS).

**Methods:**

The Wechsler Intelligence Scale for Children, fourth edition was used to divide all SeLECTS patients into two groups: patients with full‐scale intelligence quotient (FSIQ) below 80 that corresponded to cognitive impairment, and patients with FSIQ above 80 that corresponded to a normal cognitive function. The data on the resting state were recorded using magnetoencephalography. The properties of the networks were analyzed using graph theory (GT) analysis.

**Results:**

The functional connectivity (FC) of the frontal cortex in patients with FSIQ < 80 was reduced in the 12–30 Hz frequency band, and the FC of the posterior cingulate cortex was reduced in the 80–250 and 250–500 Hz frequency bands. The GT analysis showed that patients in the FSIQ < 80 group had higher strength in the 8–12 and 12–30 Hz frequency bands than those in the healthy control and FSIQ > 80 group. However, the path length was reduced in the 80–250 Hz band, and the clustering coefficient was reduced in the 12–30, 80–250, and 250–500 Hz frequency bands. Moreover, the receiver operator characteristic analysis showed that the clustering coefficient in the 12–30 and 80–250 Hz frequency bands, as well as the path length in the 80–250 Hz frequency band possessed a good discriminative ability in distinguishing the FSIQ > 80 group.

**Conclusions:**

SeLECTS patients with cognitive impairment in the early stage of the disease developed disordered networks in cognitive‐related brain regions. The clustering coefficient in the 12–30 and 80–250 Hz frequency bands as well as the path length in the 80–250 Hz frequency band might be good indicators to distinguish the cognitive impairment of SeLECTS patients at the early stage.

## Graphical abstract

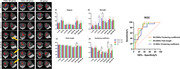

## 1 INTRODUCTION

Self‐limited epilepsy with centrotemporal spikes (SeLECTS) is the most common idiopathic focal age‐dependent epilepsy syndrome in children, accounting for approximately 15%–25% of the total pediatric epilepsy (Wirrell, 1998). The onset age in typical SeLECTS patients is usually 3–13 years, and the peak age is 7–8 years (Wirrell, 1998). A total of 75% of SeLECTS patients have seizures during the night or daytime sleep, mostly occurring shortly after falling asleep or before waking up. The duration of the seizures is short, approximately 30–120 s, and a status epilepticus may also occur. The typical symptoms are focal seizures with facial muscle twitches accompanied by several other symptoms, including numbness and paresthesia of the lips and tongue (Clemens & Majoros, 1987). The electroencephalography (EEG) background and sleep cycle are usually normal, and the interictal time is characterized by typical spikes occurring in the central temporal region, alone or in clusters, unilateral or bilateral (Clemens & Majoros, 1987).

The traditional view holds that SeLECTS patients can recover spontaneously in adulthood, and they have a good prognosis, without further neuropsychological deficits (Blom & Heijbel, 1982). However, the rapid advances in neuroscience revealed that the results of the current studies on cognitive impairment in patients with SeLECTS are still inconsistent. Some recent studies revealed that SeLECTS patients have a slightly lower full‐scale intelligence quotient (FSIQ) score compared with that in the healthy children, although still within the normal range (Galer et al., 2015; Völkl‐Kernstock et al., 2009). However, other studies showed that some SeLECTS patients have cognitive impairment at an early stage of the disease (Li et al., 2020; Yan et al., 2017), and some patients display cognitive impairment in specific areas, such as language function, memory, and reading comprehension (Currie et al., 2018; Tristano et al., 2018). However, studies on SeLECTS patients at different stages are still lacking, and therefore, it is of utmost importance to explore cognitive impairment in patients with SeLECTS at an early stage.

The research on the mechanism of cognitive impairment in SeLECTS patients has become a hot topic in recent years (Operto et al., 2019; Wickens et al., 2017; Yan et al., 2017). The pathophysiological mechanism of cognitive impairment in SeLECTS patients is complex, causing the emergence of heated discussions among scholars. A recent study using resting‐state functional magnetic resonance imaging (MRI) combined with 24 h video‐electroencephalograph (VEEG) showed that changes in brain functional network under normal background caused by frequent cortical discharge during sleep may affect the cognitive function of SeLECTS patients (Yan et al., 2017). Another study pointed out that SeLECTS patients who show continuous spike discharges during sleep are characterized by abnormalities in the functional connectivity (FC) pattern of the whole brain in the executive network and the salience network. This study revealed that the continuous spikes discharges during sleep cause the changes in the brain FC, becoming a risk of cognitive impairment for SeLECTS patients (He et al., 2020). Therefore, existing evidence suggested that cognitive impairment in SeLECTS patients was associated with changes in brain functional networks. However, the existing studies are still insufficient to explore the cognitive function of these patients in the early stage of the disease. The investigation on the relationship between functional imaging indicators and cognitive function in the early stage of the disease in such patients might not only complement existing theories but also have clinical significance.

Magnetoencephalography (MEG) is a new and noninvasive clinical imaging technique, with high temporal and spatial resolution, and can explore high‐frequency signals (Wheless et al., 2004; Xiang et al., 2010). As magnetic signals pass through tissues such as the skull and skin without attenuation, MEG has the advantage of avoiding more noise and artifact interference than EEG (Barkley & Baumgartner, 2003; Hämäläinen, 1992). Notably, MEG also has disadvantages. It is more affected by head movements than EEG, and the amplitude of the MEG signal is significantly influenced by the distance from the source, resulting in less sensitivity to deep sources than EEG (Goldenholz et al., 2009). In addition, it is less sensitive to radial currents (Baillet, 2017; Hillebrand & Barnes, 2002). Despite the evident advantages and disadvantages of MEG at present, MEG is now considered an approach with a high potential for studying brain activity thanks to the development of software analysis and advances in MEG detection.

Within this context, this study was designed and conducted to fulfill two goals. First, MEG analysis was used to construct whole‐brain FC networks to observe whether FC was specifically altered at the whole‐brain level in SeLECTS patients with cognitive impairment at the early stage of the disease. Second, the characteristics of the FC network were quantified by graph theory (GT) analysis after constructing the FC network to explore the potential existence of objective indicators of specific alterations in the topological network structure in patients with cognitive impairment at the early stage of the disease. The purpose was to explore the objective changes on functional imaging related to cognitive impairment in newly diagnosed SeLECTS patients, and to explore the relationship between cognitive impairment and neural network changes in newly diagnosed patients.

## 2 EXPERIMENTAL PROCEDURES

### 2.1 Subjects

A total of 38 children aged 6–13 years who were initially diagnosed with SeLECTS and not under any medication were enrolled at the Neurology Department of The Affiliated Brain Hospital of Nanjing Medical University and Nanjing Children's Hospital in China. All patients met the criteria of the International League Against Epilepsy (ILAE) 2017 classification for seizures (Fisher et al., 2017), and 35 of them met the inclusion criteria listed below. In addition, 18 healthy children were enrolled and used as controls. The age and gender of the children, their educational background as well as that of the parents, and family socioeconomic status were considered to maximize the avoidance of social factors as causes of differences in cognitive function.

The inclusion criteria were the following: (a) Patients who met the ILAE 2017 epilepsy syndrome classification and were diagnosed with SeLECTS. EEG showing a typical high‐amplitude spike wave originated from the central temporal region, with a normal background wave; (b) VEEG showing a high‐amplitude spike or spike‐and‐slow wave originated in the central temporal region, with normal background waves; (c) patients who were not under a therapy with antiepileptic drugs (AEDs), and aged 6–13 years; (d) patients with normal development, no other types of epilepsy, no other neurological diseases, and negative results by MRI scan; (e) parents with normal intelligence, high school education or above, with their children receiving formal education; (f) subjects and parents or legal guardians willing to sign an informed consent form as required; (g) patients without any seizure at least for 72 h before the scan and 24 h after the scan. The exclusion criteria were the following: (a) patients with metal implants in the body, including cochlear implants and cardiac pacemakers that can produce an evident noise that interferes with the test results; (b) patients with a history of other neurological or psychiatric diseases, including traumatic brain injury and schizophrenia; (c) patients with clinically significant systemic diseases; (d) children who are not expected to follow the guidelines and complete the study. The clinical details of each patient are shown in Table 1.

**TABLE 1:** Clinical patients’ data

Patients	Gender	Age (years)	Course of disease (months)	Number of seizures
1	F	6	0.4	1
2^a^	M	7	1.5	2
3	M	10	1	2
4^a^	F	6	0.9	1
5^a^	M	9	3.8	2
6	M	9	0.3	1
7	F	11	0.6	2
8^a^	F	6	1.2	3
9	M	10	0.9	1
10^a^	M	6	2.1	2
11	F	6	0.9	2
12^a^	M	7	2.9	3
13^a^	F	7	2.5	2
14	M	6	0.7	2
15	F	8	0.8	2
16^a^	M	6	1.4	2
17	F	6	0.1	1
18	M	7	0.1	1
19	M	6	0.2	1
20^a^	F	6	3.6	3
21^a^	M	7	5.5	3
22^a^	F	7	2	3
23	F	10	0.3	1
24^a^	F	6	2.1	4
25	F	7	0.3	1
26^a^	M	7	2.2	3
27	M	8	0.8	2
28^a^	F	8	3.5	5
29	M	7	1	1
30^a^	F	9	2.7	4
31	M	8	0.6	3
32	F	11	0.6	2
33^a^	M	6	3.1	3
34^a^	F	7	3.2	4
35	M	10	0.9	2

^a^ The mean of the FSIQ score is less than 80. F = female; M = male.

All children and their parents gave the informed consent to participate in the research, which was approved by the Medical Ethics Committee of Nanjing Medical University, Nanjing Brain Hospital, and Nanjing Children's Hospital.

### 2.2 MEG recordings

A whole‐head CTF275 channel MEG system (VSM MedTech Systems, Inc., Coquitlam, BC, Canada) was used to record MEG signals in a magnetically shielded room. The removal of any metal object from each subject was required before collecting the data. Then, the base of the nose and the positions 1 cm from of the ears were marked, and three coils were placed and fixed with tape. Then, the MEG data were recorded for 120 s at a sampling rate of 6000 Hz. The subjects were asked to remain still during the data recording period, gently closing their eyes, but avoiding falling asleep. At least six consecutive data fragments with a duration of 2 min were collected for each subject. The head position was determined before and after each data collection to ensure that the head movement error remained within 5 mm, otherwise, in the case of movement of more than 5 mm during the recording process, the set of data was discarded and recorded again. Data were collected for at least 3 days after the last seizure to prevent the effect of seizures on the MEG signal. In addition, electrooculography (EOG) and electrocardiography (ECG) were performed on each subject during the MEG recordings.

### 2.3 MRI scan

A 3.0‐T‐MRI (Siemens, Germany) was used for the MRI scanning of all subjects. MRI markers were placed at the position of the three magnetic circles in the previous MEG records not only to avoid the deviation in MRI caused by the change of head direction, but also to ensure that the anatomical location of MRI could be identified after the visualization of the MEG data, so that the MRI and MEG data of the patient could be accurately merged.

### 2.4 Data preprocessing

All MEG records without significant artifact segments were collected using follow‐up analysis methods. The MEG waveform signal was preprocessed through the following steps: (a) MEG data with evident artifacts (amplitude >6 pT) in the MEG waveform were excluded according to a previous research (Xiang et al., 2015); (b) the corresponding filtering processing near the 50 Hz frequency band was performed before analyzing the data to avoid the interference of the environmental AC power near the 50 Hz frequency band with the signal; (c) MEG data recording started after 3 min of recording in the empty room to ensure the collection of the background and sensor noise, and noise covariance was calculated from the source analysis; (d) heartbeat and blink events identified from ECG and EOG data were used to define projectors independently using principal component analysis. Principal components, meeting the artifact sensor topology, were manually selected and excluded using orthogonal projection (Florin & Baillet, 2015); (e) the high‐amplitude spikes of SeLECTS patients in the 1–70 Hz frequency band were identified after the screening in the previous step; (f) 60 s continuous data fragments without spikes were selected for subsequent data analysis to ensure homogeneity and the ability to conduct comparative studies due to the fact that spikes could interfere with MEG signals. Similarly, 60 s continuous data fragments from the MEG data of the eligible healthy children were selected as a control. Then, the selected MEG data were analyzed in the following seven predefined frequency bands: *δ* (1–4 Hz), *θ* (4–8 Hz), *α* (8–12 Hz), *β* (12–30 Hz), *γ* (30–80 Hz), ripple (80–250 Hz), and fast ripple (250–500 Hz).

### 2.5 The Wechsler Intelligence Scale for Children, fourth edition

The Chinese version of the Wechsler Intelligence Scale for Children, fourth edition (WISC‐IV) was used to assess the intelligence level of all subjects (WechslerDJTTCBSC, 2007) according to a previous research (Yang et al., 2013). The scores of this scale are composed of verbal comprehension index (VCI), perceptual reasoning index (Curnow et al., 2020) (PRI), working memory index (WMI), processing speed index (PSI), and FSIQ. A previous study revealed a good credibility of this scale (Baron, 2005). All the tests in WISC‐IV were completed by a clinician who is specialized in pediatric neuropsychology. The average FSIQ score of the healthy children was 90–110; 80–90 was the middle and lower range, and 70–80 was the critical range of normal FSIQ (WechslerDJTTCBSC, 2007). It is usually believed that SeLECTS children with FSIQ scores below 80 have cognitive impairment after excluding the influence of developmental disorders and neuropsychiatric diseases on cognitive function, even in the critical range (Li et al., 2020). Therefore, the SeLECTS children were divided into a cognitive impairment group and normal cognitive function group based on FSIQ = 80.

### 2.6 Functional connectivity analysis

FC at the source level was analyzed according to previous reports (Xiang et al., 2013, 2015). Accumulated source imaging was used to localize the significant neuromagnetic signals of the whole brain. First, the whole‐brain magnetic source was constructed using a wavelet‐based beamformer and the spatial resolution of 3 mm according to the algorithm described in detail in previous reports by Xiang et al. 2013, 2014, 2015). This method has been used in other related studies, and its specific effectiveness has been confirmed (Miao et al., 2019; Tang et al., 2016; Wang et al., 2010; Wu et al., 2017). Then, the above algorithm was used to calculate the virtual sensor waveform of each source, so as to perform the analysis of FC at the source level. Finally, the source neural network was estimated through the analysis of the correlation of each pair of virtual sensor signals in a 60 s time window. In other words, the statistical analysis on the relationship between the virtual sensor signals from the dual‐source pair was performed, and the relationship between each pair of signals was determined by the calculation of the correlation factor or the correlation coefficient. The relevant factors are defined as follows:

(1) $$\;R_{\left(X_{a},X_{b}\right)}=\frac{C\left(X_{a},X_{b}\right)}{SX_{a}SX_{b}}$$

where $R_{\left(X_{a},X_{b}\right)}$ represents the correlation of the source pair at two positions (*a* and *b*). $X_{a}$ and $X_{b}$ represent the signals from two sources, which are computationally connected in pairs. $C(X_{a},X_{b})$ represents the average of the two source signals. $SX_{a}$ and $SX_{b}$ represent the standard deviations of the two source signals.

Exemplary FC networks are shown in Figure 2, where the blue arrow represents the negative connection, and the red arrow represents the positive connection. Positive connections indicate positive correlations among brain regions, whereas negative connections indicate negative correlations among brain regions. $T_{P}$ represents the *t*‐value of the correlation. Thresholds were used as checkpoints to identify significant connections. The $T_{P}$ of all source pairs was calculated to determine the connection threshold. The relevant factors are defined as follows:

(2) $$\;T_{P}=R\sqrt{\frac{K-2}{1-R^{2}}}$$

whereR represents the correlation of the source pair, andKrepresents the number of data points connected. The $T_{P}$ value corresponding to a *p* value <.01 was used in this study as the threshold to obtain the FC network. In summary, the correlation measure was quantified and corrected with GT analysis to detect FC. The issues regarding the field spread and volume conduction were solved using two‐step beamforming and statistical assessments.

### 2.7 Graph theory analysis

A graph in GT analysis is composed of many nodes connected by lines. GT analysis was used to quantitatively analyze the characteristics of the FC network of the SeLECTS patients and healthy control (HC) individuals with the methods previously published (Wu et al., 2017). In this study, the magnetic source was the node in the network, and the functional connection was the edge connecting the nodes. Then, the average of the network parameters for each frequency band was calculated, such as the clustering coefficient, the degree, the path length, and the connection strength. Notably, all the network parameters are average over nodes.

#### 2.7.1 Strength

The connection strength ($S_{i}$) of the nodei is the sum of the weight values of the edges directly connected to it in a weighted network G (Reijneveld et al., 2007),

(3) $$S_{i}=\frac{1}{N}\sum_{i\;=\;1}^{N}w_{ij}$$

whereas the strength of the network ($S_{A}$) is defined as the average of the connection strength of all nodes:

(4) $$\;S_{A}=\frac{1}{N}\sum_{i\;=\;1}^{N}S_{i}$$

#### 2.7.2 Degree

The degree of a node *i* is the number of nodes directly connected to it. Hence, it is often used to evaluate the importance of a node in the network. In a weighted network G containing *N* nodes and *k* edges, the network degree ($D_{A}$) is the average of the degrees of all nodes in the network:

(5) $$\;D_{A}=\frac{1}{N}\sum_{i\;=\;1}^{N}d_{i}$$

where $D_{A}$ indicates the development and compliance of the network (Rubinov & Sporns, 2010; Wang et al., 2016).

#### 2.7.3 Path length

The path length between two nodes, namely, *i* and *j*, is the sum over the edges length along the path connecting these two points; the length of each edge is equal to the inverse of the weight value of the edge, that is, 1/$w_{ij}$ (Reijneveld et al., 2007). The average value of the path length of all node pairs in the network is the average path length $L_{A}$ of the network:

(6) $$L_{A}=\frac{1}{N\left(N-1\right)}\sum_{i,j}L_{i,j}$$

$\mathrm{where}\;L_{A}$ reflects the global characteristics of the network. $L_{i,j}$ is the shortest distance between node *i* and node *j*. This definition assumes that $L_{i,j}$ = 0 if node *i* cannot be reached by node *j* or if *i* = *j*.

#### 2.7.4 Clustering coefficient

The clustering coefficient is an index indicating the local aggregation degree of nodes in a graph. A more natural definition of clustering coefficient is the fraction of triangles that are closed over the total possible number. It is defined as follows: assuming that a node sends *k* edges, the possible maximum number of edges between the nodes (*k*) connected by these *k* edges is *k* (*k* − 1)/2. Then, the clustering coefficient of this node is the score value obtained by dividing the actual number of edges by the maximum number of edges (Reijneveld et al., 2007). The clustering coefficient of the node *i* for a weighted network G containing *N* nodes and *k* edges is determined as the average geometric weight of all nodes:

(7) $$C_{i}=\frac{1}{di\left(d_{i}-1\right)}\sum_{j,k\in Gj,k\neq i}{\left(w_{ij}\cdot w_{jk}\cdot w_{ki}\right)}^{1/3}$$

where *d*i is the number of edges that the node *i* is directly connected to (the degree of the node *i*), and *w* is the weight of the edges that connect the two nodes. The clustering coefficient ($C_{A}$) of the network G represents the average of the clustering coefficient of all nodes in the network:

(8) $$C_{A}=\frac{1}{N}\sum_{i\;=\;1}^{N}C_{i}$$

$\mathrm{where}\;C_{A}$ reflects the local characteristics of the network.

The above data analysis was performed using the MEG Processor software (https://sites.google.com/site/braincloudx/).

### 2.8 Statistical analysis

Statistical analysis was performed using SPSS 24.0 (SPSS Inc., Chicago, IL, USA). The distribution of the main functional connections of the three groups of experimental subjects was located and measured by Fisher's exact probability method and chi‐square tests. The clinical data between the two SeLECTS patient groups were compared using the independent samples *t*‐test. The data distribution was evaluated by the Kolmogorov–Smirnov test, one‐way analysis of variance was performed on the four network parameters, and the homogeneity of variance test was also performed. The correlation between network parameters and clinical characteristics in each frequency band was evaluated using Pearson or Spearman correlation analysis. The *p* value was corrected by Bonferroni multiple comparison method, using *p* < .05 as the threshold for statistically significant difference. In detail, 3 groups of subjects and 7 frequency bands were present in the FC analysis, so the resulting *p*‐values needed to be corrected 21 times. Three groups of subjects, 7 frequency bands, and 4 parameters were present in the GT analysis, so the obtained *p*‐values needed to be corrected 84 times. The *p*‐values shown in Section 3 were obtained after Bonferroni correction. In addition, one‐way analysis of variance revealed the differences among the three groups; the four network parameters with significant differences between the FSIQ < 80 and FSIQ > 80 groups were also determined using binary logistic regression analysis to determine the diagnostic value of the FSIQ < 80 group through the use of the receiver operator characteristic (ROC) curve, and the area under curve (AUC) was plotted to investigate the diagnostic performance.

## 3 RESULTS

### 3.1 Subjects

The data from 35 SeLECTS patients and 18 HCs were included in this study. The patient group was composed of 17 patients with FSIQ < 80 and 18 patients with FSIQ > 80. The mean age of all the SeLECTS patients was 7.51 ± 1.60 years, and the mean age of the HC group was 7.56 ± 1.38 years. The details are summarized in Table 1.

### 3.2 Clinical data

**FIGURE 1:**
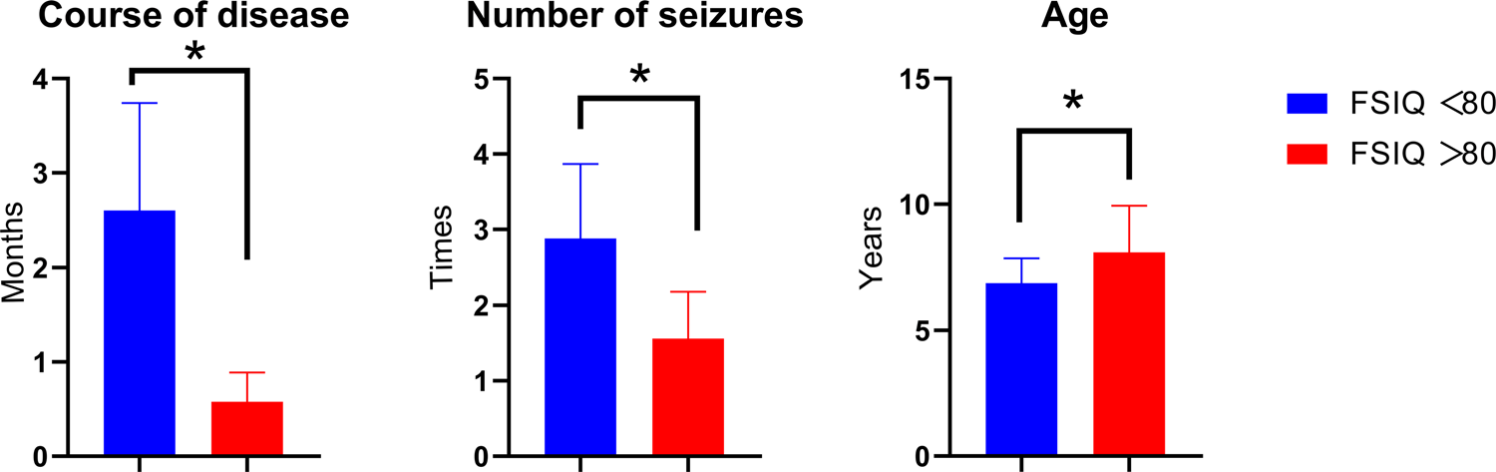
Comparison of the course of the disease, age, and the number of seizures between the full‐scale intelligence quotient (FSIQ) > 80 and the FSIQ < 80 group. *p < .05

The mean course of the disease in the FSIQ < 80 group was 2.60 ± 0.58 months, the mean course of the disease in the FSIQ > 80 group was 0.58 ± 0.31 months, and the difference was statistically significant (*p* = .0001) (Figure 1). The mean number of seizures in patients in the FSIQ < 80 group was 2.88 ± 0.99, whereas it was 1.56 ± 0.62 in the patients in the FSIQ > 80 group, and the difference was statistically significant (*p* = .0001) (Figure 1). The age of the two groups of patients was also compared. The mean age of the patients in the FSIQ < 80 group was 6.88 ± 0.99, the mean age of the patients in the FSIQ > 80 group was 8.11 ± 1.84, and the difference was statistically significant (*p* = .02) (Figure 1).

### 3.3 WISC‐IV scores of the SeLECTS group and control group

The WISC‐IV scores in the two groups of SeLECTS patients were the following: FSIQ = 85.69 ± 11.06, VCI = 84.03 ± 9.63, PRI = 84.94 ± 13.97, WMI = 89.86 ± 12.03, and PSI = 87.97 ± 15.04. The five WISC‐IV scores in the entire group of SeLECTS patients were significantly lower than those of the HC group (*p* < .001). However, no significant difference was found in the age of all the SeLECTS patients group compared to the age of the HC group. The detailed WISC‐IV scores of the HC group and the two groups of SeLECTS patients are shown in Table 2.

**TABLE 2:** Wechsler Intelligence Scale for Children, fourth edition (WISC‐IV) scores in the full‐scale intelligence quotient (FSIQ) < 80 group, FSIQ > 80 group, and healthy controls (HC)

WISC‐IV	FSIQ < 80 group	FSIQ > 80 group	HC group
VCI	79.18 ± 4.88	88.61 ± 10.83	110.61 ± 9.85
PRI	73.76 ± 7.79	95.50 ± 9.51	106.39 ± 11.74
WMI	82.00 ± 7.82	97.28 ± 10.59	105.83 ± 8.57
PSI	77.35 ± 7.10	98.00 ± 13.63	97.39 ± 10.30
FSIQ	76.41 ± 2.35	94.44 ± 8.50	108.00 ± 8.55

Abbreviations: PRI, perceptual reasoning index; PSI, processing speed index; VCI, verbal comprehension index; WMI, working memory index.

### 3.4 Functional connectivity network analysis

**FIGURE 2:**
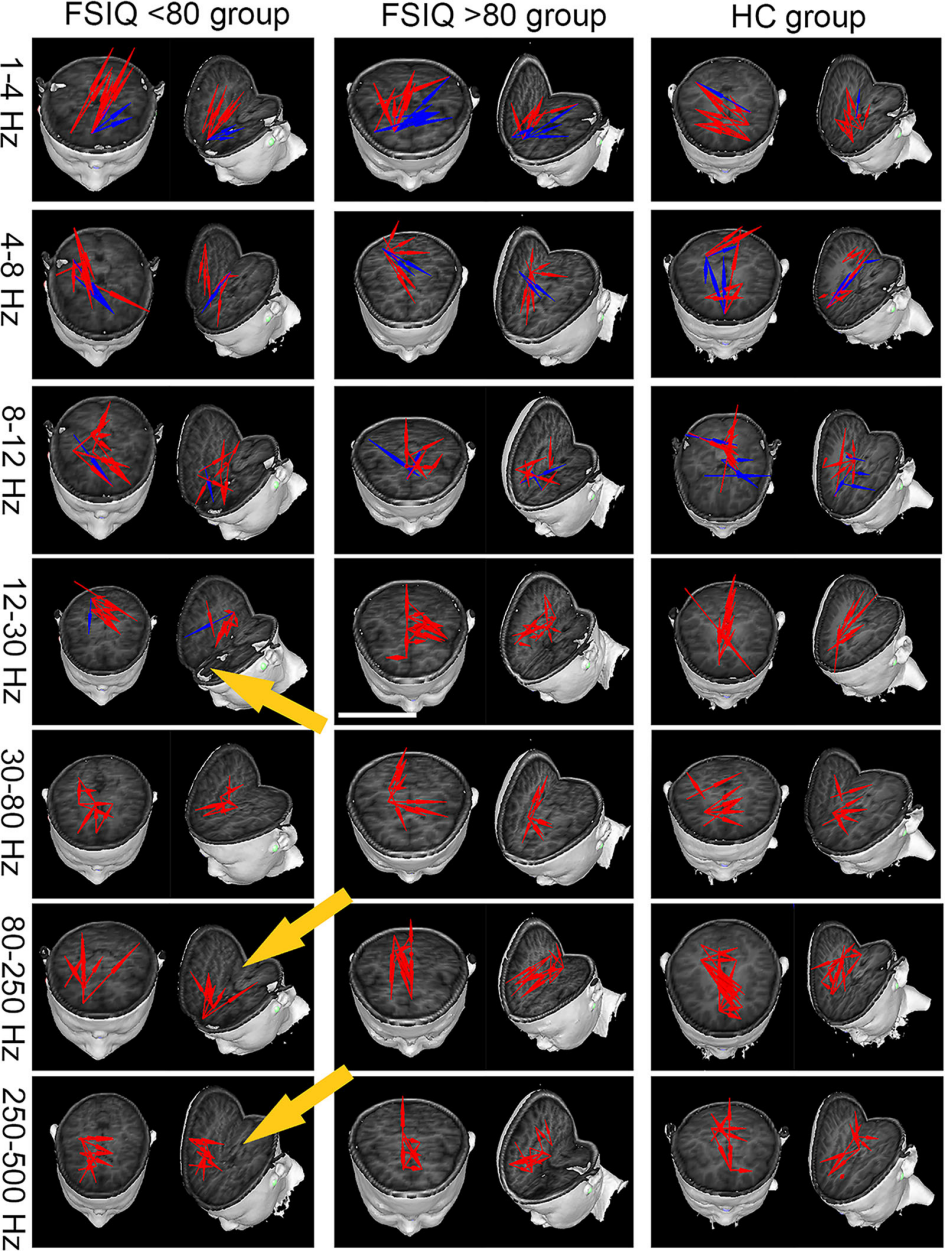
Three groups of typical functional connectivity (FC) network images in seven frequency bands. The red arrows in the figure represent positive functional connections, and the blue arrows represent negative functional connections. The yellow arrow points out the brain area where the FC network of the full‐scale intelligence quotient (FSIQ) < 80 group was significantly abnormal compared with the FSIQ > 80 group and the healthy control (HC) group. No significant difference was found in the FC network of the FSIQ > 80 group compared to the HC group.

The FC network showed a specific pattern in different frequency bands. Both positive and negative FC were detected in this study. The differences in FC distribution across frequency bands among the three groups of subjects detected during our observations were mainly in the positive FC, whereas the differences in the negative FC were not significant. Patients in the FSIQ < 80 group had significantly reduced positive functional connections in the frontal cortex in the 12–30 Hz band and significantly reduced positive functional connections in the posterior cingulate cortex (PCC) region in the 80–250 and 250–500 Hz bands than the FSIQ > 80 group and HC group. No significant difference in the FC network was found in the FSIQ > 80 group compared to the HC group (see Table 3 for details). The typical FC network patterns among the three groups are shown in Figure 2.

**TABLE 3:** Abnormal network locations in the full‐scale intelligence quotient (FSIQ) < 80 group compared to the FSIQ > 80 group and healthy controls (HC)

Frequency band (Hz)	Abnormal network location	Reduced/increased	ANOVA (*p*‐value)	A vs. B (*p*)	B vs. C (*p*)	A vs. C (*p*)
12–30	Frontal cortex	Reduced	.001	.002	.57	.001
80–250	Posterior cingulate cortex	Reduced	.001	.001	.61	.002
250–500	Posterior cingulate cortex	Reduced	.002	.008	.32	.001

Abbreviations: A, FSIQ < 80 group; ANOVA, analysis of variance; B, FSIQ > 80 group; C, HC group.

### 3.5 Graph theory analysis

**FIGURE 3:**
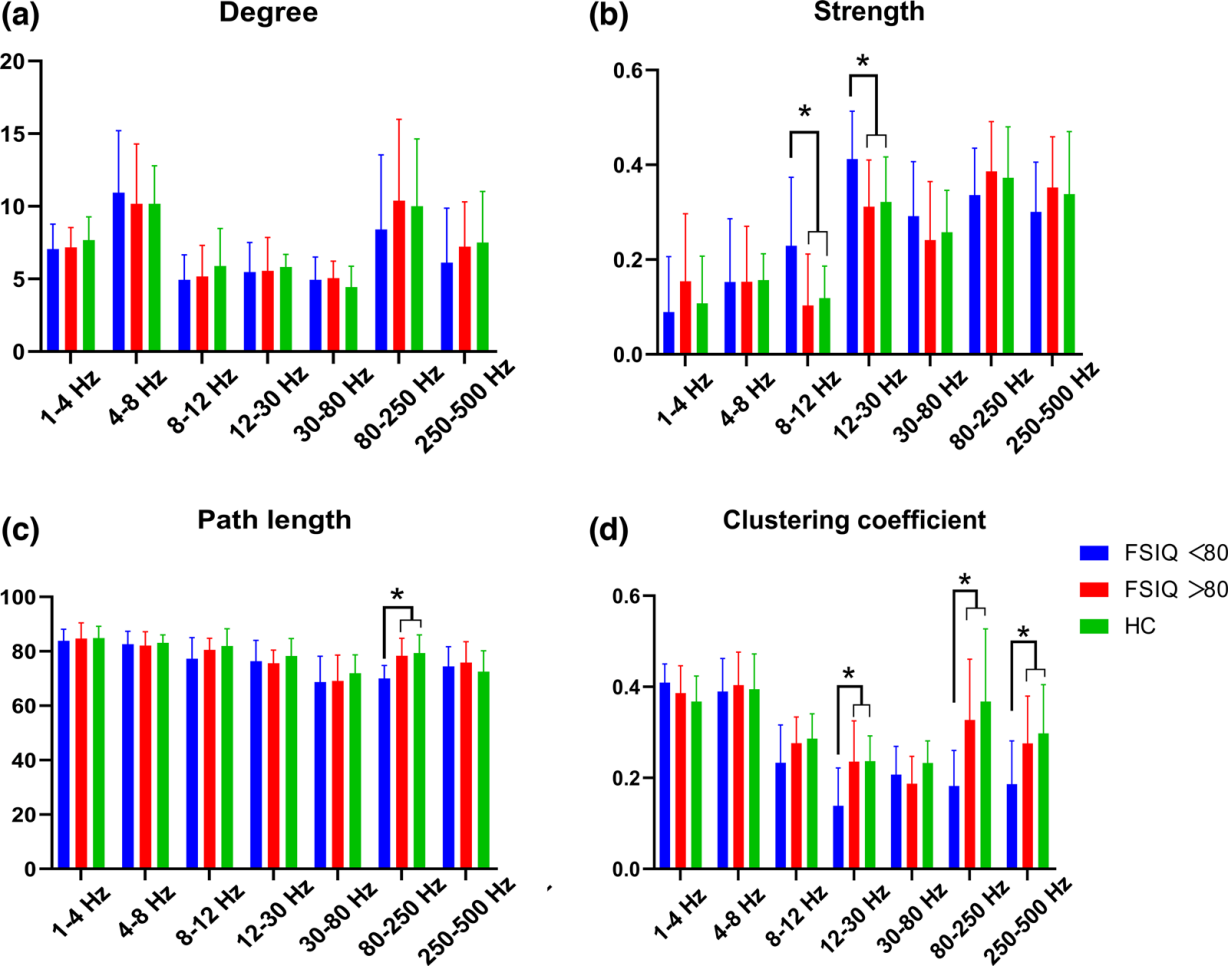
Differences in four graph theory (GT) network parameters (degree, path length, strength, and clustering coefficient) among the healthy control (HC) group, the full‐scale intelligence quotient (FSIQ) > 80 and the FSIQ < 80 group at multiple frequency bands. *p < .05

The GT analysis showed no significant differences in degree in the three groups in each frequency band. The strength in the 8–12 and 12–30 Hz frequency bands in the FSIQ < 80 group was significantly increased than that in the HC group and FSIQ > 80 group. However, the clustering coefficient of the FSIQ < 80 group was significantly reduced in the 12–30 Hz frequency band than that in the HC group and FSIQ > 80 group. The path length in the 80–250 Hz frequency band in the FSIQ < 80 group was significantly reduced than that in the HC group and FSIQ > 80 group, and the clustering coefficient was also significantly reduced. The clustering coefficient in the 250–500 Hz frequency band in the FSIQ < 80 group was significantly reduced than that in the HC group and FSIQ > 80 group (see Table 4 for details). The network parameters of the FSIQ > 80 group were not significantly different from those in the HC group. The results of the GT analysis among the three groups are shown in Figure 3.

**TABLE 4:** Abnormal network parameters in the full‐scale intelligence quotient (FSIQ) < 80 group compared to the FSIQ > 80 group and healthy controls (HC)

Frequency band (Hz)	Abnormal network parameters	Reduced/increased	ANOVA (*p*‐value)	A vs. B (*p*)	B vs. C (*p*)	A vs. C (*p*)
8–12	Strength	Increased	.003	.001	.64	.005
12–30	Strength	Increased	.007	.004	.76	.009
12–30	Clustering coefficient	Reduced	.003	.001	.97	.001
80–250	Path length	Reduced	.002	.001	.69	.001
80–250	Clustering coefficient	Reduced	.001	.002	.95	.001
250–500	Clustering coefficient	Reduced	.006	.013	.52	.002

Abbreviations: A, FSIQ < 80 group; ANOVA, analysis of variance; B, FSIQ > 80 group; C, HC group.

### 3.6 Logistic regression analysis

**FIGURE 4:**
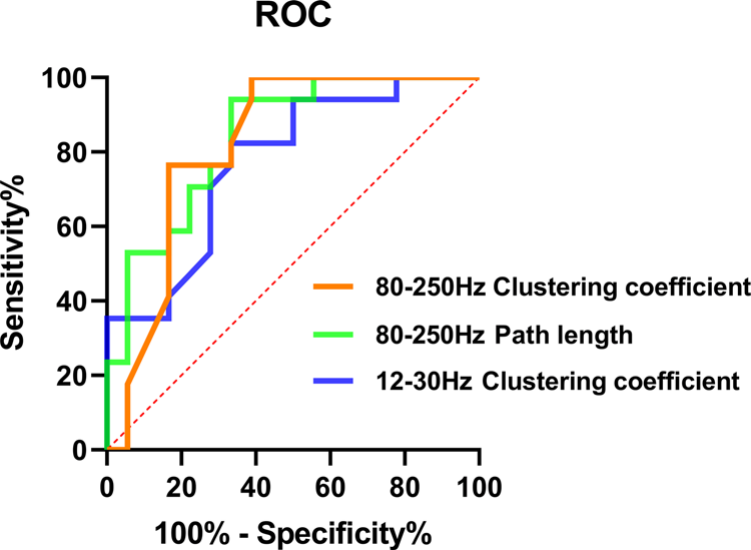
Receiver operator characteristic (ROC) curves of the clustering coefficient in the 12–30 and 80–250 Hz frequency bands, as well as the path length in the 80–250 Hz frequency band to discriminate self‐limited epilepsy with centrotemporal spikes (SeLECTS) individuals with full‐scale intelligence quotient (FSIQ) < 80

Binary logistic regression analysis was used to evaluate the four network parameters that were significantly different between the FSIQ < 80 and FSIQ > 80 groups by one‐way analysis of variance, in order to identify predictors for cognitive impairment in patients with SeLECTS at an early stage. Notably, the binary regression analysis using FSIQ < 80 as the dependent variable indicated that the clustering coefficient (95% CI [.67, .97]; *p* = .001) and the path length (95% CI [.71, .97]; *p* = .006) at the 80–250 Hz frequency band and the clustering coefficient (95% CI [.62, .93]; *p* = .006) at the 12–30 Hz frequency band were statistically significant. The ROC curves for the three parameters are shown in Figure 4. Details on the AUC area, sensitivity, specificity, and accuracy values are shown in Table 5.

**TABLE 5:** Metrics for logistic regression models

Indicators	AUC	Sensitivity (%)	Specificity (%)	Accuracy (%)
12–30 Hz, clustering coefficient	0.77	70.59	72.22	70.6
80–250 Hz, path length	0.84	70.59	77.78	71.4
80–250 Hz, clustering coefficient	0.82	76.47	83.33	76.5

Abbreviation: AUC, area under the curve.

### 3.7 Correlation analysis

The correlation between the abnormality of GT parameters in each frequency band of the patient brain networks was analyzed, as well as the age, the course of epilepsy, and the number of seizures of the corresponding patients, and no evident statistically significant results were found.

## 4 DISCUSSION

In this work, MEG was used to explore whole‐brain FC network patterns in SeLECTS patients with early cognitive impairment, and network characteristics were quantified using GT analysis to explore the objective FC network alterations associated with cognitive impairment. Imaging markers that might help suggest early cognitive impairment were evaluated. To our knowledge, this is the first time that resting‐state MEG has been used to study changes in brain network patterns in SeLECTS patients with cognitive impairment. Thus, the present study contributed to the understanding of early changes in brain network patterns in SeLECTS patients with cognitive impairment and improved the differentiation between patients with and without cognitive impairment at the early stage of the disease.

### 4.1 Clinical data

Patients in the FSIQ < 80 group had a longer disease course, more seizures, and a younger age of onset compared to the other group with FSIQ < 80. Seizures can destroy the structure of the brain tissue and cause abnormal brain metabolism. It can be predicted that, in the long run, the damage to the brain caused by repeated seizures is irreversible (Johnson, 2019). However, only limited evidence is available proving that seizures in patients with SeLECTS lead to brain damage or injury. Furthermore, it is not easy to understand the cause of brain dysfunction in the patient from the seizure and the underlying etiology of epilepsy. Previous studies suggested that the neuropsychological deficits in patients with epilepsy are related to the age at the onset (O'Leary et al., 1983). Various neuropsychological tests revealed that patients with an onset at a younger age have worse performance than those with an onset at an older age (O'Leary et al., 1983). The present study found that the onset age of the SeLECTS patients with cognitive impairment was significantly lower than that of patients with normal cognitive function, which was in agreement with the results of previous studies. This might be due to the fact that the anatomical function network matures in childhood, and SeLECTS also develops during this period, thus affecting the normal development of the cognitive function (Massa et al., 2001). Although the cognitive impairment was detected in these patients, they were still in the early stages of the disease; thus, the claim that spikes and seizures contribute to cognitive decline in children should be taken with caution. Our hypothesis was that the epileptic activity and cognitive impairment might be the result of underlying common pathological mechanisms: for example, genetic factors (Shi et al., 2020; Xiong & Zhou, 2017) or specific features of an underlying disease (Parisi et al., 2017). For example, current studies reported that poor cognitive function was considered to be an important characteristic of atypical benign epilepsy with centrotemporal spikes (Fejerman, 2009). Further increase in the size of the sample and studies with a longer follow‐up are needed to confirm these results.

### 4.2 Functional connectivity network analysis

The default mode network (DMN) is a functional network of the brain, which is usually more active in a passive state (at rest) than during task execution (Lin et al., 2016). A previous study suggested that DMN is related to cognitive function (Chen et al., 2018). The DMN can be divided into two parts at a functional level: An anterior part that is centered on the medial frontal cortex, and a posterior part that is centered on the PCC (Raichle, 2015). Moreover, the frontal cortex and PCC area are the core brain areas for the DMN network (Maldjian et al., 2014). In‐line with previous findings (Ofer et al., 2018), this study revealed a decrease in FC in the anterior and posterior DMN regions in the SeLECTS children with cognitive impairment, suggesting the presence of a disorder in the DMN network in these patients with cognitive impairment. It was currently suggested that cognitive impairment and interictal epileptiform activity might be distinct symptoms of DMN dysfunction (Yan et al., 2017). However, it was equally likely for this present study that both network dysfunction and cognitive impairment as well as SeLECTS epileptiform activity could be manifestations of the underlying disease (Gong et al., 2021; Parisi et al., 2017). As the SeLECTS patients enrolled in this study were patients with a primary diagnosis, caution was required when speculating that seizures and spike–wave discharges contributed to network dysfunction. Our hypothesis was that FC network dysfunction might have a precursor role, occurring prior to cognitive impairment as well as epileptiform activity. Further confirmation of the relationship between network dysfunction and cognitive impairment in SeLECTS patients is still needed in the future through additional longitudinal follow‐up studies with expanded sample sizes.

As regards lower frequency bands, FC in the frontal cortex at the 12–30 Hz frequency band in the FSIQ < 80 group was weak. Substantial evidence suggests that beta oscillations play a key role in regulating brain function (Varela et al., 2001). A study found that beta oscillations are often associated with active inhibition (Buzsáki & Draguhn, 2004). In the process of cognition, beta oscillation supports perceptually relevant activity within sensorimotor and frontoparietal cortices (Engel & Fries, 2010). Therefore, our speculation was that children with SeLECTS with cognitive impairment might lack the inhibition of task‐independent networks, which could be a sign of suboptimal network coordination. The frontal lobe is a key area of the brain related to global cognitive functions, especially executive functions (Douw et al., 2011). This suggested that weakened FC in brain regions responsible for cognitive function could lead to cognitive impairment in some patients.

As regards the higher frequency bands, the PCC area at the 80–500 Hz frequency band in the patients of the FSIQ < 80 group was weak. Previous studies revealed that high‐frequency oscillations in the network behave as an extension of the epileptic network (Chaitanya et al., 2015; Jacobs et al., 2012; Velmurugan et al., 2018). However, this study found that patients with cognitive impairment in the early stage of the disease had weakened FC in the PCC region, which was not in agreement with previous studies. These children had a short disease course and few seizures because the subject of this study was in the early stage of the disease. A potential explanation could be that the diminished connectivity of brain regions associated with cognitive function in the high‐frequency bands might be a precursor feature of SeLECTS patients with cognitive impairment. The FC in the higher frequency bands gradually progresses in the direction of an extended network as the disease progresses. Further follow‐up studies are still needed to confirm this in the future.

### 4.3 Graph theory analysis

The GT analysis showed that patients in the FSIQ < 80 group had higher strength in the 8–12 and 12–30 Hz frequency bands than that in the HC and the FSIQ > 80 group. However, the path length was reduced in the 80–250 Hz band, and the clustering coefficient was reduced in the 12–30, 80–250, and 250–500 Hz frequency bands, suggesting that the brain network in the SeLECTS patients with early cognitive impairment presented an under‐optimized topology pattern. However, no significant difference in the network mode was observed between the SeLECTS patients with normal cognitive function and healthy children.

The present study showed that strength increased significantly in the SeLECTS patients with cognitive impairment. Our speculation was that patients with cognitive impairment had abnormal connections in their brain networks at the early stage of the disease, and these connections were over‐enhanced. Over‐connection self‐modifies the connection over time in children with a normal growth and development of the brain, finally developing into a properly connected and mature brain network (Supekar et al., 2009). If the modification mechanism of the connection is damaged during development or if the disease prevents adequate pruning, it may result in an excessive connectivity (Ye et al., 2014). An over‐connected network reduces the efficiency of the effective information processing, increases the threshold of brain excitement, and causes cognitive impairment (Wu et al., 2017). However, whether self‐correction occurs to this over‐enhanced connectivity in SeLECTS patients during growth and development as in normal children needs further confirmation by additional follow‐up longitudinal studies.

To our knowledge, this study was the first using GT analysis to extract abnormal network parameters to determine the diagnostic value of SeLECTS patients with cognitive impairment at the early stage of the disease. Our results showed that the reduced clustering coefficient in the 12–30 and 80–250 Hz frequency bands, as well as the reduced path length in the 80–250 Hz frequency band showed good sensitivity and specificity, and the accuracy rate was high. This result underlined the disordered topological network structure in SeLECTS patients with cognitive impairment at the early stage of the disease. The clustering coefficient in the 12–30 Hz band and the path length and clustering coefficient in the 80–250 Hz band might be good indicators to distinguish the cognitive impairment of SeLECTS patients in the early stage. The path length represents the shortest distance between pairs of nodes in the network, and the decrease in the average path length reflects the abnormal increasing trend of integration in the global FC network (Ismail & Karwowski, 2020). The clustering coefficient represents the likelihood of connectivity among node neighbors, and the decrease in the average clustering coefficient reflects a decreasing trend in local integration in FC networks (Reijneveld et al., 2007). Taken together, SeLECTS children with cognitive impairment in the early stage of the disease exhibited a randomized network pattern characterized by reduced average path length and reduced average clustering coefficient, indicating that the network is efficient at transferring information but is not robust (Ismail & Karwowski, 2020). This randomized network with reduced average clustering coefficient and average path length was also reported in studies of medial temporal lobe epilepsy and Alzheimer's disease (Liao et al., 2010; Stam et al., 2009), and this disruption of network function is linked to cognitive impairment (Li et al., 2020; Liao et al., 2010). The generally accepted view is that a disordered network structure may be the final result of different types of brain injury caused by the disappearance of the original neurons and connections and the random creation of new connections (Stam et al., 2009). However, the reasons for the disordered network patterns in these patients still need a careful exploration due to the very limited evidence on brain or nerve damage in SeLECTS patients. Moreover, it is still uncertain whether this brain dysfunction is transient or reversible, and further follow‐up studies should be performed to confirm it.

Overall, the GT analysis revealed that SeLECTS patients with cognitive impairment in the early stage of the disease exhibited a disordered FC network pattern. However, it is still not clear whether the occurrence of this disordered network pattern was before or with SeLECTS. Moreover, the relevant reports are scarce and still lacking sufficient evidence. A recent study showed that children with SeLECTS have increased cognitive assessment scores 1 year after the treatment, and their MEG results suggested that the normalization of DMN abnormalities in children with SeLECTS induced by AEDs may explain the improvement in cognitive function (Niu et al., 2021). This evidence suggests that the abnormal FC network in the early stage of SeLECTS may be reversible, and that the FC network may be able to normalize if seizures were controlled. However, follow‐up studies with a larger sample size are still needed to determine whether abnormal FC network is normalized with the increase of age.

A correlation between abnormal network parameters and clinical data was not found in patients in the FSIQ < 80 group. This could be potentially due to the small sample size, or because the relationship between the two factors was not a simple linear relationship. Therefore, these results should be confirmed by further studies.

### 4.4 Limitations

Although this study found some exciting new results, some limitations should be underlined. First of all, the sample size of this study was small. A larger sample size might allow the finding of more potential differences between groups. The goal of the next work is to include larger samples to confirm and complement the present experimental conclusions. Second, this study only used FSIQ as the main basis for grouping the patients. Thus, more subtest results should be included in our further studies to evaluate the pathophysiological mechanism of the changes in cognitive function in different fields in a more detailed and systematic approach. Finally, although the visible artifacts and noise in MEG results were removed as better as possible, some of them were still difficult to remove, and this limitation might be solved with the progress of the analysis technology in the future.

## 5 CONCLUSIONS

This study showed that the interictal FC network in SeLECTS patients with cognitive impairment at the early stage of the disease had specific differences in specific frequency bands. SeLECTS patients with cognitive impairment had a longer course, more seizures, and younger age than those in patients with normal cognitive function. SeLECTS patients with cognitive impairment in the early stage of this disease had disordered network patterns in cognitive‐related brain areas such as the frontal cortex and PCC. The brain network of SeLECTS patients with early cognitive impairment showed an under‐optimized topology pattern. The clustering coefficient in the 12–30 and 80–250 Hz frequency bands, as well as the path length in the 80–250 Hz frequency band, might be good indicators to distinguish the cognitive impairment of SeLECTS patients in the early stage.

## CONFLICT OF INTEREST

The authors state that they have no conflict of interest. They also confirm that they have read the statement of the journal on ethical publishing and that this report is consistent with those guidelines.

### PEER REVIEW

The peer review history for this article is available at https://publons.com/publon/10.1002/brb3.2830

## Data Availability

The data that support the findings of this study are available on request from the corresponding author. The data are not publicly available due to privacy or ethical restrictions.
